# Peripheral basophil reactivity, CD203c expression by Cryj1 stimulation, is useful for diagnosing seasonal allergic rhinitis by Japanese cedar pollen

**DOI:** 10.1002/iid3.69

**Published:** 2015-06-26

**Authors:** Yoshimasa Imoto, Tetsuji Takabayashi, Masafumi Sakashita, Takahiro Tokunaga, Takahiro Ninomiya, Yumi Ito, Norihiko Narita, Takechiyo Yamada, Shigeharu Fujieda

**Affiliations:** Department of Otorhinolaryngology Head and Neck Surgery, Faculty of Medical Sciences, University of FukuiFukui, Japan

**Keywords:** allergic rhinitis, basophil, CD203c, IgE

## Abstract

Measuring specific IgE can yield direct, accurate, and objective data. Nevertheless, clinical symptoms of allergy are often inconsistent with these data. Recently, the expression of CD203c, a surface marker of basophils, has been reported as capable of distinguishing allergic patients. This study compared specific IgE in serum and skin tests against antigen to assess CD203c as a biomarker correlated with allergic rhinitis (AR). We asked 3,453 subjects whether they experienced any AR related symptom. All subjects were assessed for six specific IgEs for common aeroallergens. Skin tests were also conducted for six aeroallergens. We observed the reactivity of peripheral basophil by measuring the levels of CD203c by Cryj1 stimulation using flow cytometry. Of the 3,453 participants, 1,987 (57.5%) possessed Japanese cedar pollen (JCP) specific IgE in their serum. Among those 1,987 JCP specific IgE positive participants, 552 (27.8%) had not experienced any allergic symptom during the JCP season. The levels of CD203c in the peripheral basophil by Cryj1 stimulation were significantly higher in SAR-JCP subjects than in non-SAR-JCP subjects (Cryj1 0.5 ng/ml: 2.25 ± 0.90% vs. 60.2 ± 27.4%, *p* < 0.01, Cryj1 50 ng/ml: 1.89 ± 0.90% vs. 68.0 ± 21.2%, *p* < 0.01). Our results indicate that the levels of CD203c in peripheral basophils by Cryj1 stimulation is a more objective and reliable marker that better reflects the allergic reaction by SAR-JCP in vivo than measuring specific IgE in serum or skin tests.

## Introduction

Seasonal allergic rhinitis (SAR) by Japanese cedar (*Cryptomeria japonica*) pollen (SAR-JCP), the most common allergic disease in Japan, is an important public health issue because of its high prevalence, its effects on the general public, and its associated medical expenses 1. We reported previously that the prevalence of AR was 44.2% in adults, and that the most common allergen in AR is JCP (89.6%) in Japan 2. The diagnostic tools used for AR are positive history of nasal symptoms by allergen exposure, positive reaction of skin test, and nasal provocation test 3. Although skin tests are useful and easy to perform, they sometimes cause an anaphylactic reaction, and/or a false positive or negative reaction. Specific IgE in serum is also useful to clarify the immunological reaction with accuracy and objectivity, but the specific IgE in serum and allergic symptoms are not always detected together. Therefore, SAR-JCP diagnosis requires the development of more accurate and reliable examination tools.

Although basophils exist as only 1% of circulating whole blood cells, they play fundamentally important roles in allergic reactions. They express high-affinity IgE receptors (Fc∊RI) as well as mast cells. IgE binds to Fc∊RI, which is present on the surface of basophils. This complex can bind to an antigen, leading to basophil activation and degranulation, releasing histamine, and producing IL-4 and IL-13, which are crucially important for the development of an allergic reaction 4. Basophils express various surface markers, some of which are useful as markers of basophil activation. Actually, CD63, which is known as a lysosome-associated membrane glycoprotein (LAMP-3), belongs to the transmembrane-4 superfamily. When basophils are stimulated by antigen via Fc∊RI, CD63 is expressed on the basophil surface. Recent reports have described that up-regulation of CD63 might reflect an anaphylactic phase 5. However, CD203c, which is also present in basophils, is a glycosylated type 2 transmembrane family that belongs to the ectonucleotide pyrophosphatase/phosphodiesterase enzyme family 5,6. Actually, CD203c is expressed constitutively on basophil surface membranes with lower concentration, which is up-regulated by allergen stimulation. These activation mechanisms suggest that CD203c is related to piecemeal degranulation 7–9. The reaction of piecemeal degranulation lasts longer than anaphylactic degranulation by slow and persistent mediator release without crosslinking of Fc∊RI by antigen 10,11. These mechanisms might contribute to the pathogenesis of chronic allergic inflammation. Özdemir et al. 12 reported that a CD203c expression pattern clearly discriminated patients with pollen allergy from healthy subjects with high sensitivity and specificity. Complementary components also play crucially important roles in allergic inflammation. Anaphylatoxins C3a and C5a are involved in asthma pathogenesis 13. It had been reported that the levels of C3a and C5a in bronchoalveolar lavage (BAL) fluid were elevated in asthmatic subjects after allergen challenge 14. Furthermore, the levels of C3a and C5a in serum are increased in acute exacerbations of asthma 15.

For this study, we asked 3,453 participants whether they experienced any allergic rhinitis (AR) related symptoms and measured six inhalant-aeroallergen-specific IgEs. Skin tests were also conducted for the six aeroallergens. Results show that the levels of CD203c in peripheral basophils by Cryj1 stimulation were significantly higher in the SAR-JCP group than in non-SAR-JCP groups, irrespective of the results of IgE and skin test. Furthermore, the levels of C3a and C5a in serum were significantly higher in SAR-JCP group than in non-sensitized group. Our results suggest that the responsiveness of peripheral basophils, the levels of CD203c by Cryj1 stimulation, might be a more objective and reliable marker reflecting the allergic reaction by SAR-JCP in vivo than specific IgE in serum and skin test.

## Methods

### Subjects

Between 2003 and 2011, 3,453 hospital workers and university students were invited to participate in an epidemiological survey of AR. Total and specific IgE (Japanese cedar, *Dermatophagoides*, *Dactylis glomerata*, *Ambrosia artemisiifolia*, *Candida albicans*, and *Aspergillus*) were measured using the Immuno CAP method (Pharmacia Diagnostics AB, Uppsala, Sweden). They were asked whether they had experienced nasal symptoms related to AR. We also invited 50 randomly selected people to participate in this study during March 2011 and 2012, when subjects were exposed to Japanese cedar pollen. The 50 participants were divided into four groups according to AR symptoms, results of Immuno CAP, and skin test against JCP: Group I, specific IgE negative, skin test negative without SAR-JCP symptoms; Group II, specific IgE positive, skin test negative without SAR-JCP symptoms; Group III, specific IgE positive, skin test positive without SAR-JCP symptoms; Group IV, specific IgE positive, skin test positive with SAR-JCP symptoms. The participants’ physical characteristics are presented in Table 2. No participant had been treated using any medicine such as histamine H1 receptor antagonists, oral corticosteroids, or intranasal spray of corticosteroids. All participants provided written informed consent to participate in the study. The study was approved by the ethical committees of the University of Fukui, Japan.

### Skin test

We performed intradermal skin tests against JCP allergen using an allergen extract (Torii Pharmaceutical Co. Ltd., Tokyo, Japan). Subjects were injected with 20 μl of allergen fluid in the forearm. Then the diameter of flare and wheal were measured 15 min after the injections. Subjects exhibiting flare of skin of >20 mm or wheal >9 mm were considered “positive” against JCP.

### Nasal symptom scores

The numbers of sneezes, numbers of bouts of nasal blowing, and nasal congestion during JCP season were recorded for all 50 participants. Based on that information, the participants were graded on a scale of Japanese Rhino-conjunctivitis Quality of Life Questionnaire (JRQLQ) followed by Japanese Guideline for allergic rhinitis 16.

### Measuring levels of CD203c in peripheral basophils

Whole blood was collected with heparin during the JCP season. Reportedly, basophils are distinguishable from other cells using three-color flow cytometry to detect cells that are CD3^−^, CRTH2^+^, and CD203c^+^ 17. Actually, CRTH2, also called CD294, is expressed on basophils, eosinophils, and Th2 cells 18. The levels of CD203c in basophils were measured by Allergenicity Kit (Beckman Coulter, CA). We used Cryj1 as a major allergen of JCP. Whole blood with heparin was incubated at two Cryj1 concentrations (0.5 ng/ml, 50 ng/ml) for 15 min. Anti-IgE antibody at 8 μg/ml as a positive control and PBS as a negative control were used for stimulation. The levels of CD203c on basophils were determined using the fluorescence of negative control and positive control. PC7-conjugated anti-CD3, FITC-conjugated anti-CRTH2, and PE-conjugated anti-CD203c antibodies were added and analyzed by BD FACSCalibur (BD Bioscience, San Diego). At least 500 basophils were detected at each assay.

### Measuring anaphylatoxins in serum

Venous blood was collected during the JCP season. Samples were then centrifuged at 4°C. Supernatants were stored at −80°C until use. Subsequently, C3a and C5a enzyme-linked immunoassays (ELISAs) were performed according to the manufacturer's instructions (BD Biosciences, San Diego).

### Statistical methods

All data are reported as the median. Differences between groups were analyzed using unpaired *t*-tests. A *p*-value of less than 0.05 was considered statistically significant.

## Results

**Figure 1:**
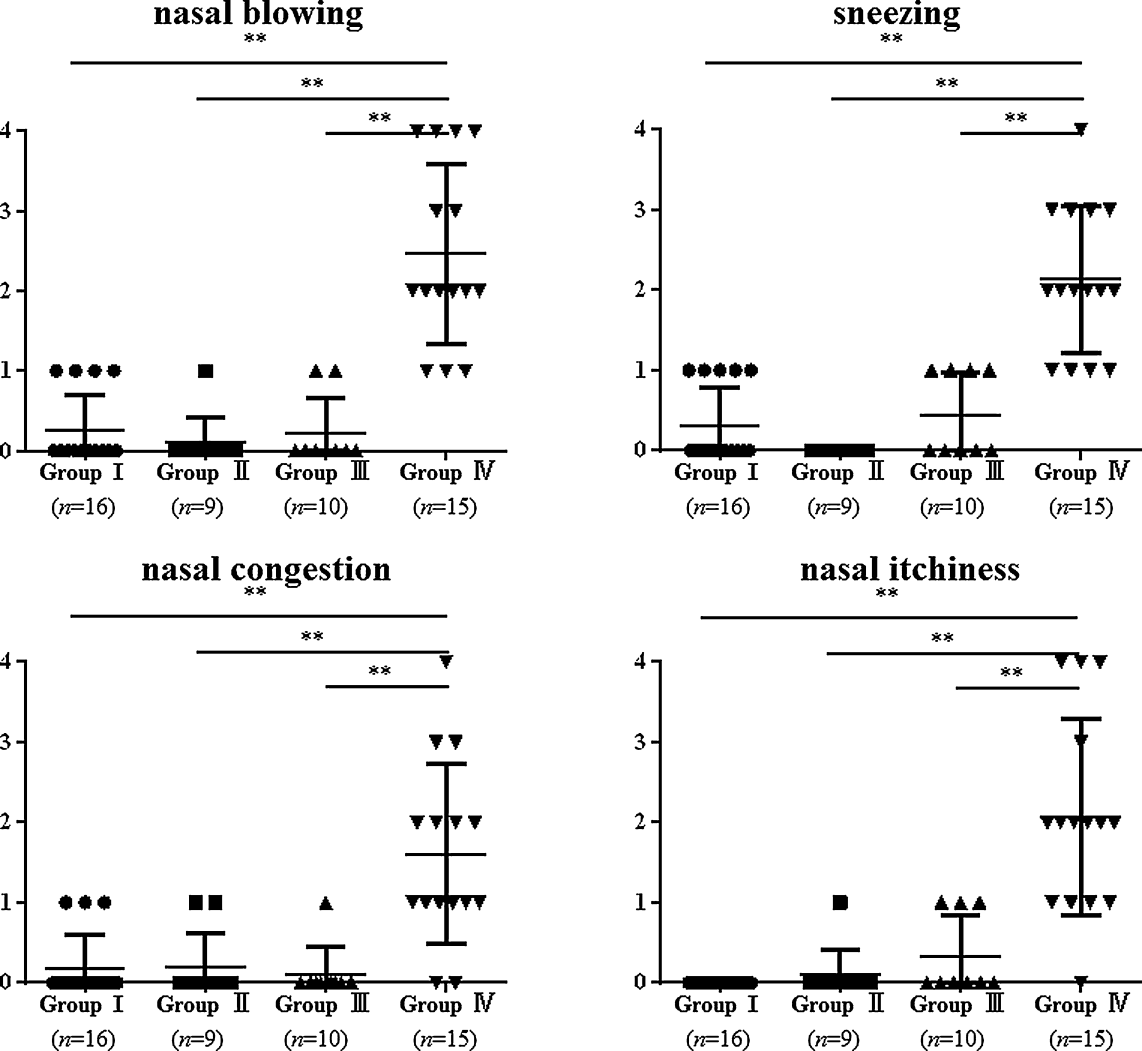
Symptom scores. Nasal symptom scores (nasal blowing, sneezing, nasal congestion, nasal itchiness) in SAR-JCP subjects (group IV) were significantly higher than both sensitized (groups II and III) and control subjects (group I). **p < 0.01.

For this epidemiological survey of AR, 3,453 volunteers were recruited. Subject characteristics are presented in Table1. In this study, subjects showing Immuno CAP score ≥2 were defined as sensitized. The most common airborne allergen is JCP, and sensitized ratios of the six inhalant aeroallergens are the following: JCP (57.5%), dust mites (39.6%), *D. glomerata* (24.4%), *A. artemisiifolia* (9.9%), *C. albicans* (5.6%), and *Aspergillus* (2.1%) (Table1). These results are in line with those reported from an earlier study 2. Among the 1,987 subjects of the JCP sensitized group, 1,435 (72.2%) subjects had experienced seasonal allergic symptoms during March–April, when Japanese cedar disperse, but 552 subjects (27.8%) had not experienced SAR-JCP symptoms. To ascertain the differences between JCP sensitized subjects with and without allergic symptoms during JCP season, subjects were divided into four groups according to the AR symptoms, results of Immuno CAP, and results of skin tests for JCP (Table2). We assessed the nasal symptom score during the JCP season. SAR-JCP subjects (group IV) revealed high nasal symptom scores (sneezing 2.20 ± 0.86, nasal congestion 1.60 ± 1.06, nasal blowing 2.47 ± 1.13, nasal itchiness 2.2 ± 1.15) that were higher than those of the other three groups (Fig. 1).

**Table 1:** Characteristics of the study population (3,453 subjects)

Characteristics	
Sex (male:female)	1172:2281
Mean age ± SD	30.3 ± 12.4
Total IgE, geometric mean (IU/ml)	89.0 ± 4.2

Specific IgE (UA/ml)	Sensitization (range, UA/ml)
*Cryptomeria japonica* pollen	57.5% (<0.34–100)
Dermatophagoides	39.6% (<0.34–100)
*Dactylis glomerata*	24.4% (<0.34–100)
*Ambrosia artemisiifolia*	9.9% (<0.34–39)
*Candida albicans*	5.6% (<0.34–49.2)
*Aspergillus* spp.	2.1% (<0.34–28.9)

**Table 2:** Characteristics of CD203c study subjects

	Control (group I)	Sensitized (group II)	Sensitized (group III)	SAR-JCP (group IV)
Sex (male:femele)	8:8	2:8	5:4	7:8
Age (y), mean ± SD	26.1 ± 3.9	23.9 ± 5.4	28.1 ± 7.8	27.9 ± 5.1
Skin test	(−)	(−)	(+)	(+)
Total IgE (IU/ml) mean (range)	34.2 (5–460)	60.3 (26–200)	94.1 (8–340)	173.8 (29–8700)
JC specific IgE (IU/ml)	<0.3	1.4	6.7	18.3
Dermatophagoides specific IgE (IU/ml)	<0.3	0.9	1.4	1.2
Dactylis glomerata specific IgE (IU/ml)	<0.3	0.4	0.9	4.9
Ambrosia artemissfolia specific IgE (IU/ml)	<0.3	0.3	0.5	0.6
Candida albicans specific IgE (IU/ml)	<0.3	<0.3	<0.3	<0.3
Aspergillus spp specific IgE (IU/ml)	<0.3	<0.3	<0.3	<0.3

**Figure 2:**
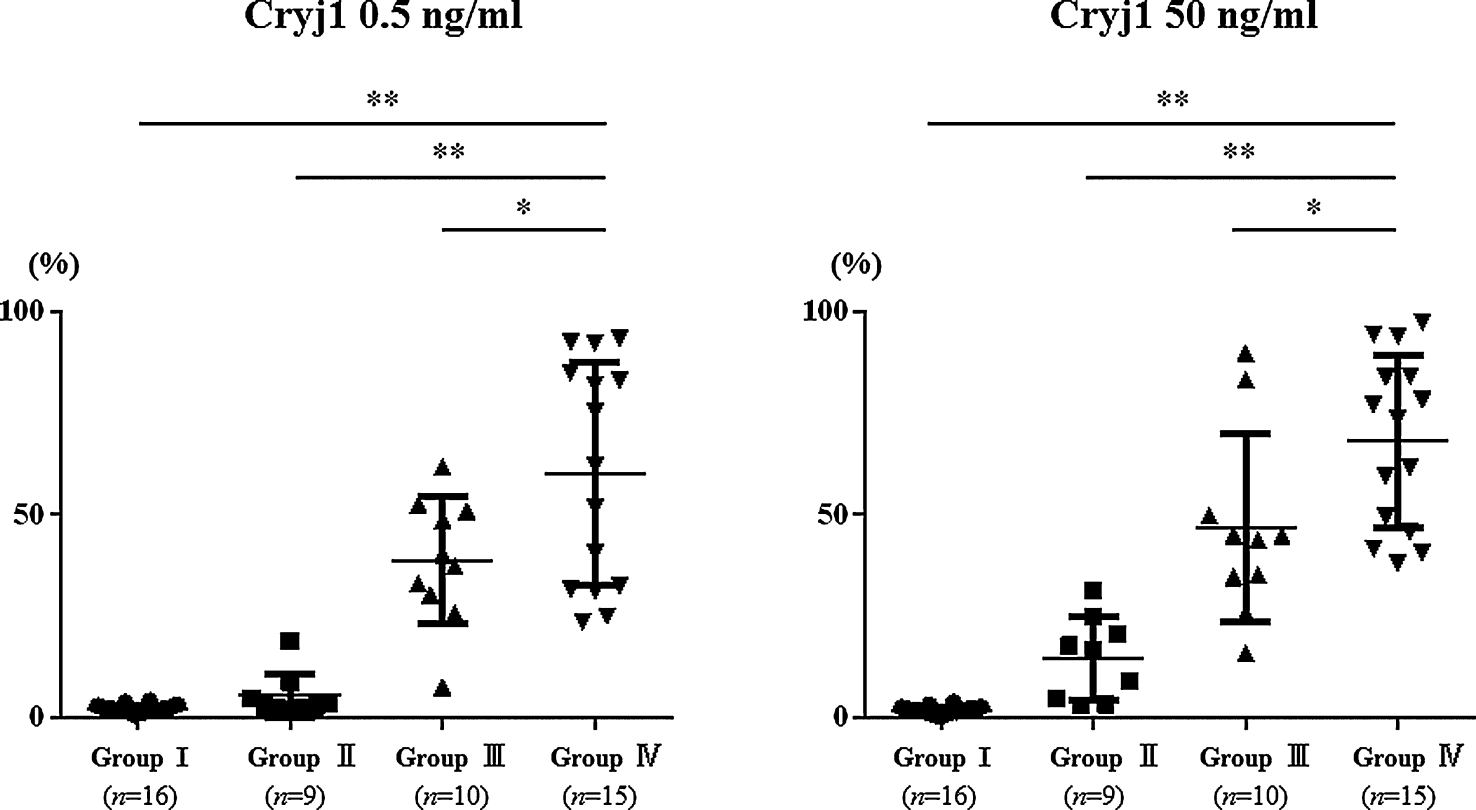
Levels of CD203c in basophils against JCP. Levels of CD203c in control group (group I) showed low activation ratio (0.5 ng/ml, 0.3–3.8%; 50 ng/ml, 0.4–3.8%). Those of sensitized subjects showed negative reaction in skin test (group II) caused lower levels of CD203c (0.5 ng/ml, 2–8.8%; 50 ng/ml, 3.1–31.2%). SAR-JCP group (group IV) showed moderate to high upregulation of CD203c (0.5 ng/ml, 23.6–93.4%; 50 ng/ml, 38.1–97.5%). Group III showed moderate levels of CD203c (0.5 ng/ml, 25.6–61.8%; 50 ng/ml, 25.6–89.9%). *p < 0.05, **p < 0.01.

To assess the responsiveness of peripheral basophil for JCP stimulation, we conducted basophil activation tests using JCP extract (Cryj1). The levels of CD203c in peripheral basophils by Cryj1 stimulation were significantly higher in group IV (*p* < 0.05) than in any of the three other groups (Fig. 2). Although, clinically, skin testing is the most reliable examination to distinguish SAR-JCP to date 16, group III subjects showed no SAR-JCP symptoms in spite of positive skin tests. This result suggests that a positive result on a JCP skin test does not invariably distinguish patients as SAR-JCP. The levels of CD203c in peripheral basophils by Cryj1 stimulation in group IV were significantly higher than in group III subjects (Cryj1 0.5 ng/ml: 38.8 ± 15.7% vs. 60.2 ± 27.4%, *p* < 0.05, Cryj1 50 ng/ml: 46.8 ± 23.3% vs. 68.0 ± 21.2%, *p* < 0.05) (Fig. 2). Therefore, measuring CD203c in peripheral basophils by Cryj1 stimulation might be a more reliable examination than skin testing for SAR-JCP diagnosis. Most importantly, group II (specific IgE positive, skin test negative without SAR-JCP symptoms) showed quite low levels of CD203c in peripheral basophils by Cryj1 stimulation than group IV (SAR-JCP subjects) did, indicating that not all people with JCP-specific IgE will present symptoms of SAR-JCP. Another component that was not JCP specific IgE in serum might be involved in representing symptoms of SAR-JCP.

**Figure 3:**
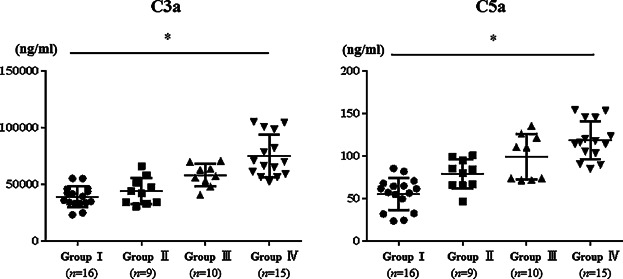
Results of C3a and C5a in serum. Mean concentrations of C3a are the following: 38917.6 ± 9393.6 ng/ml in group I, 48705.4 ± 10981.1 ng/ml in group II, 50232.2 ± 13346.5 ng/ml in group III, and 64785.1 ± 20136.7 ng/ml in group IV. Significant difference was found between groups I and IV. Regarding C5a, the mean concentrations are as follows: 63.4 ± 18.4 ng/ml in group I, 83.4 ± 18.1 ng/ml in group II, 93.5 ± 24.6 ng/ml in group III, and 124.1 ± 46.0 ng/ml in group IV. A significant difference was also found between groups I and IV. *p < 0.05.

The complement components C3a and C5a play crucially important roles in the development of allergic inflammation 13. We next examined the concentrations of serum anaphylatoxins C3a and C5a. Concentrations of both anaphylatoxins were significantly higher in group IV (SAR-JCP) (64785.1 ± 20136.7 ng/ml, 124.1 ± 46.0 ng/ml, respectively) than in group I (38917.6 ± 9393.6 ng/ml, 63.4 ± 18.4 ng/ml, respectively) (*p *< 0.05). Although not significantly, the concentrations of both anaphylatoxins were higher in group IV (SAR-JCP) than in group II (specific IgE positive, skin test negative without SAR-JCP symptoms) and group III (specific IgE positive, skin test positive without SAR-JCP symptoms) (Fig. 3) (group II vs. group IV, C3a: *p* = 0.0670, C5a: *p* = 0.0832, group III vs. group IV, C3a: *p* = 0.090, C5a: *p* = 0.131, respectively). These results suggest that the concentrations of anaphylatoxins influence the SAR-JCP symptoms exactly.

## Discussion

The current study revealed that the most common aeroallergen is JCP (1,987 of 3,453 participants (52.7%) were sensitized by JCP); however, 27.8% of sensitized subjects had not experienced any SAP-JCP symptoms, even during the JCP season. We also showed that the levels of CD203c in peripheral basophils by JCP stimulation yielded more objective and more specific data about allergic reactions by comparing skin tests and specific IgE in serum. Both mast cells and basophils possess Fc∊RI on the cell surface. Both cause allergic inflammation after crosslinking of surface-bound IgE by antigen. Different from mast cells, basophils are obtainable from whole peripheral blood cells. Recent reports in the relevant literature describe that basophil activation reflects pathological and clinical conditions in some allergic diseases 9,19.

**Figure 4:**
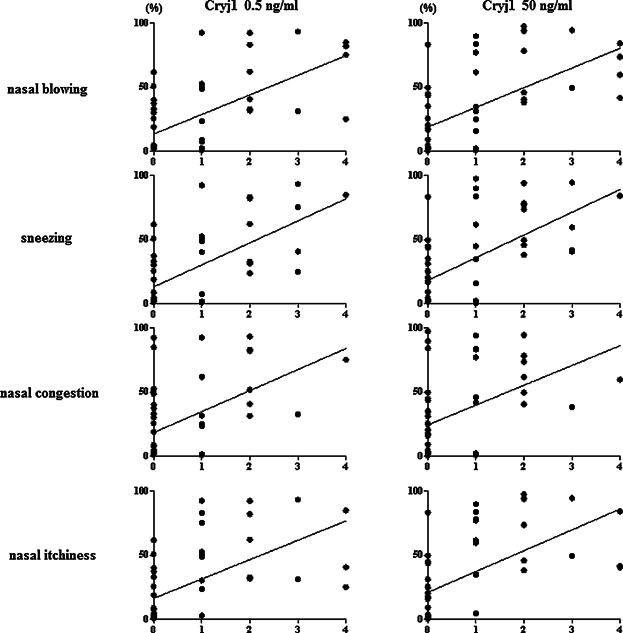
Correlations between the levels of CD203c and nasal symptom scores. There are significant correlations between the levels of CD203c and each nasal symptom scores. Cryj1 0.5 ng/ml; sneezing: *R*^2^ = 0.343, *p* < 0.0001, nasal blowing: *R*^2^ = 0.344, *p* < 0.0001, nasal congestion: *R*^2^ = 0.220, *p* < 0.001, nasal itchiness: *R*^2^ = 0.316, *p* < 0.0001. Cryj1 50 ng/ml; sneezing: *R*^2^ = 0.313, *p* < 0.0001, nasal blowing: *R*^2^ = 0.330, *p* < 0.0001, nasal congestion: *R*^2^ = 0.176, *p* < 0.001, nasal itchiness: *R*^2^ = 0.330, *p* < 0.0001.

Often, CD63 is used as a basophil activation marker because CD63 expression is associated more closely with degranulation. However, several studies have dissociated the appearance of CD63 from histamine release. Actually, CD203c is expressed on resting basophils at low concentrations; its expression is rapidly up-regulated following activation. Measuring CD203c expression in basophils might be more consistent with the AR symptoms and pathology than measuring CD63. Reportedly, the level of CD203c expression is correlated significantly with nasal symptoms among SAR patients 20. We also demonstrated the correlations between the levels of CD203c and nasal symptoms and found the significant correlations: (Cryj1 0.5 ng/ml sneezing: *R*^2^ =0.343, *p* < 0.0001, nasal blowing: *R*^2^ = 0.344, *p* < 0.0001, nasal congestion: *R*^2^ = 0.220, *p* < 0.001, nasal itchiness: *R*^2^ = 0.316, *p* < 0.0001) (Cryj1 50 ng/ml sneezing: *R*^2^ = 0.313, *p* < 0.0001, nasal blowing: *R*^2^ = 0.330, *p* < 0.0001, nasal congestion: *R*^2^ = 0.176, *p* < 0.001, nasal itchiness: *R*^2^ = 0.330, *p* < 0.0001). Our current data are consistent with those presented in an earlier report (Fig. 4).

Perhaps the most interesting finding from this study is that basophil activation test is a more specific examination than measuring specific IgE in serum or skin tests (Fig. 2). This result indicates that merely possessing antigen specific IgE alone is insufficient to develop allergic symptoms or the mechanism by which allergic inflammation between skin and nasal mucosa might be different. Regarding venom hypersensitivity, Korosec et al. 21 reported that basophil activation test had significantly higher diagnostic sensitivity than skin tests. Another component might be involved in representing SAR-JCP symptoms.

Complementary components have an important role of innate immune system against bacteria and other pathogens. They form immune complex with surface antigen by “pattern recognition molecules.” Then they start attacking foreign bodies by mediating the variety of inflammatory responses 22,23. C3a and C5a are generated not only via the classic IgG/antigen immune-complex pathway; β-tryptase in human mast cells can also generate bioactive C3a and C5a 24. Basophils possess receptors for C3a and C5a. The binding of anaphylatoxins to these receptors can engender the release of chemical mediators such as histamines, LTC4, IL-4, and IL-13 25,26. The present study showed that although not significant, concentrations of both anaphylatoxins, C3a and C5a, were higher in group IV (SAR-JCP) than those in group II (JCP sensitized, skin test negative without SAR-JCP symptoms) or group III (JCP sensitized, skin test positive without SAR-JCP symptoms) (Fig. 3). These results suggest that anaphylatoxins are involved in representing of SAR-JCP symptoms through basophil activation. Our most recent data showed significantly lower concentrations of both anaphylatoxins in patients with sublingual immunotherapy against JCP (submitted data). Serum anaphylatoxins might play a crucially important role in SAR-JCP development through basophil activation. Additional studies must be conducted to ascertain the precise contribution of anaphylatoxins to basophil activation inducing SAR-JCP.

A member of interleukin-1 family, IL-33, is a ligand for ST2 (IL-33Rα) 27. IL-33 is expressed constitutively in epithelial and endothelial cells. It can be released by necrotic cells and injured cells, which act as “alarmin” in vivo 28,29. According to a great deal of recent reports, IL-33 plays a crucially important role for developing allergic diseases 30,31. Basophils express ST2, which is up-regulated by IL-3 32, and which can be stimulated by IL-33 to produce Th2 cytokines without cross-linking of Fc∊RI 33,34. However, IL-33 also prompts basophils to secrete IL-4, IL-13, and IL-8 with IL-3 and/or Fc∊RI activation, which accelerate Fc∊RI-induced mediator release 32. We reported previously that serum concentrations of IL-33 were significantly higher in patients with SAR-JCP than in controls 35. These results support the inference that the concentration of IL-33 affects basophil reactivity or symptoms of SAR-JCP. In the present study, we did not analyze the levels of IL-3 and ST2. To emphasize the effects on the up-regulation of CD203c, we consider that more investigations are required.

Among sensitized subjects with positive skin test reaction in our study, four subjects exhibited AR related nasal symptom and diagnosed as SAR-JCP during the next pollen season. They showed moderate to high CD203c expression as well as SAR-JCP subjects (Cryj1 0.5 ng/ml: 48.7–61.8%, Cryj1 50 ng/ml: 25.6–89.9%) (Table3). These results may indicate that up-regulation of CD203c reflect the onset of AR. To verify the prediction of the onset of AR, we consider that further investigations are needed about sensitized subjects.

**Table 3:** Characteristics of sensitized subjects who developed SAR-JCP during the next pollen season

Sex	Skin test	JC specific IgE (IU/ml)	CD203c (Cryj1:0.5 ng/ml)	CD203c (Cryj1:50 ng/ml)
M	+	89.9	52.5	34.6
M	+	50	50.9	25.6
F	+	4.35	61.8	83.3
M	+	1.14	48.7	89.9

In conclusion, results show that in SAR-JCP group, responsiveness of peripheral basophils, the levels of CD203c by Cryj1 stimulation are significantly higher than those of non-SAR-JCP groups (irrespective of possessing JCP-specific IgE in serum or skin test positive). Furthermore, although not statistically significant, concentrations of both anaphylatoxins were increased in one group (SAR-JCP) compared with the other three groups (non-SAR-JCP groups). Our results imply that peripheral basophil reactivity, CD203c expression by Cryj1 stimulation can be a useful examination tool for the diagnosis of SAR-JCP and that serum anaphylatoxins might be involved in the development of SAR-JCP through basophil activation.
